# Trust in information, political identity and the brain: an interdisciplinary fMRI study

**DOI:** 10.1098/rstb.2020.0140

**Published:** 2021-04-12

**Authors:** Adam Moore, Sujin Hong, Laura Cram

**Affiliations:** ^1^ Department of Psychology, University of Edinburgh, 7 George Square, Edinburgh EH8 9JZ, UK; ^2^ Neuropolitics Research Lab, School of Social and Political Science, University of Edinburgh, 18 Buccleuch Place, Edinburgh EH8 9LN, UK

**Keywords:** trust, identity, functional magnetic resonance imaging (fMRI), media and information source

## Abstract

Misinformation has triggered government inquiries and threatens the perceived legitimacy of campaign processes and electoral outcomes. A new identity polarization has arisen between Remain and Leave sympathizers in the UK Brexit debate, with associated accusations of misinformation use. Competing psychological accounts of how people come to accept and defend misinformation pit self-reinforcing motivated cognition against lack of systematic reasoning as possible explanations. We harness insights from political science, cognitive neuroscience and psychology to examine the impact of trust and identity on information processing regarding Brexit in a group of Remain identifiers. Behaviourally, participants' affective responses to Brexit-related information are affected by whether the emotional valence of the message is compatible with their beliefs on Brexit (positive/negative) but not by their trust in the source of information. However, belief in the information is significantly affected by both (dis)trust in information source and by belief compatibility with the valence of the information. Neuroimaging results confirm this pattern, identifying areas involved in judgements of the self, others and automatic processing of affectively threatening stimuli, ultimately supporting motivated cognition accounts of misinformation endorsement.

This article is part of the theme issue ‘The political brain: neurocognitive and computational mechanisms’.

## 1. Introduction

There is increasing concern about social media's magnification of misinformation, misleading information and conspiracy theories (hereafter misinformation) and its impact on democracy (e.g. [1–3]; for reviews, see [4,5]). Psychological accounts of misinformation acceptance differ as to whether effortful cognition is primarily involved in the acceptance or the rejection of misinformation. Accounts on the acceptance end of the spectrum (self-reinforcing (SR) accounts) propose that misinformation acceptance is largely the product of alignment with an individual's preferred worldview/ideology/prior beliefs. When such alignment occurs, individuals may selectively attend to and process information compatible with what they already believe [6–8]. Alternatively, they may selectively search for perceived weaknesses in belief-incompatible information to dismiss it, even at the expense of truth ([9]; cf. [10]). Effortful rejection (ER) accounts propose that misinformation is typically accepted on the basis of superficial characteristics and shallow processing (cf. [11]), but when reasoning is engaged, people can reliably discriminate fake from real news regardless of ideological compatibility ([12]; though right-wing conservatives show greater susceptibility to misinformation than left-wing liberals—[13,14]; but see [15]). A recent identity-based hybrid (I-bH) account proposes that contextually salient goals control where effort is deployed, with self/identity/ideology-reinforcement weighed against desires for epistemic accuracy [16]. This model posits key roles for orbitofrontal cortex (OFC) and ventromedial prefrontal cortex (vmPFC) in computing competing goals and the value of differing beliefs, respectively, which can then bias downstream processing.

Neuroimaging evidence is broadly compatible with these accounts. Areas associated with affect/emotion, value computation and conflict processing, such as lateral and medial OFC and anterior cingulate cortex (ACC), show increased activity when political partisans are confronted with information potentially damaging to their preferred candidate [17] and when trying to control affective reactions while explicitly evaluating socially relevant stimuli (e.g. abortion, murder; [18]). Similarly, vmPFC is more active when pre-existing beliefs bias reasoning performance [19] and such activity predicts increasing belief in given information [20]. This is consistent with the I-bH model, and to some extent with SR frameworks. By contrast, and supporting both ER and I-bH accounts, disbelieving information activates distributed brain regions involved in effortful cognition, including the left inferior frontal gyrus (IFG), bilateral anterior insula (AI), dorsal ACC extending to superior frontal gyrus (SFG), and superior parietal lobe [20], while right lateral PFC activation corresponds to correct logical inference in the face of competing beliefs [19].

However, this research does not systematically disentangle the affective impact of the belief (in)compatibility of information itself versus the (dis)trust of the source of that information. In other words, how do people respond when (in)compatible information is delivered via a (dis)trusted source? This is critical because source identity can impact information acceptance regardless of actual content (e.g. [21]), and such identity cues can trigger motivated cognition ([8]; see also [22]). However, the affective impact of belief-incompatible information (without source identity) can drive a disconnection with external information and increased processing in default-mode networks [23]. Here we investigate how information source and belief compatibility *combine* to affect judgements of belief and feelings about politically salient information. Neuroimaging is useful in disentangling predictions made by different frameworks: SR accounts predict that trusted sources and compatible information should result in greater belief and greater activation in areas associated with effortful cognition (e.g. lateral PFC, ACC), and a mismatch between (dis)trusted source and (in)compatible information may elicit effortful cognition related to decreased belief (i.e. motivated rejection). ER accounts predict less activity in these areas in response to trusted/compatible stimuli, but possibly more in response to mismatch stimuli (which potentially provide cues triggering reasoning processes), though it is unclear what relation effortful reasoning may have to belief in this case. Under the I-bH account we can assume that our participants are motivated in large part, though not completely, by identity protection concerns related to their self-identification as Remain voters (on which they were pre-screened; see Methods). This suggests predictions largely in line with SR accounts, though vmPFC activity related to judging various beliefs (identity protection and truth/accuracy) against one another may emerge.

## 2. Methods

### (a) Participants

Thirty-eight, right-handed, UK national, native English-speaking participants aged 18–42 (mean = 25.5, 20 females) underwent blood oxygenation level-dependent (BOLD) contrast fMRI scanning. Three were excluded owing to failure to record button responses. All were Brexit Remain supporters familiar with social media platforms. Pre-screening was conducted separately to avoid affecting behaviour. All participants provided informed consent and were paid £20. A debrief followed the fMRI scans. The study was approved by the Research Ethics Committees of the School of Social and Political Sciences and the Research Ethics procedures of the Edinburgh Imaging facility at The Queen's Medical Research Institute (EIF-QMRI) at the University of Edinburgh.

### (b) Stimuli

We extracted text from tweets related to Brexit (posted between 1 January 2019 and 14 August 2019) gathered from various media sources, and separately we pre-screened all of our participants to determine a bespoke list of their three most-used and least-used media sources (based on the British social attitudes 2018 questionnaire, Q216, (https://www.bsa.natcen.ac.uk/media/39286/questionnaire_2018_v2.pdf), and an open-choice option; see electronic supplementary material, table S1). In the initial stage, two reviewers rated tweet texts as Negative, Positive or Ambiguous. From the Negative and Positive rated texts, we selected short texts, preferably one-line tweets and, where necessary, edited these to remove source information. The remaining tweets were validated in a pilot experiment by separate Remain-voting participants (i.e. not the fMRI sample; electronic supplementary material, Methods, figure S2; A full list of 120 tweet texts (60 negative, 60 positive) used in the present study is given in electronic supplementary material, table S4).

**Figure 1.**
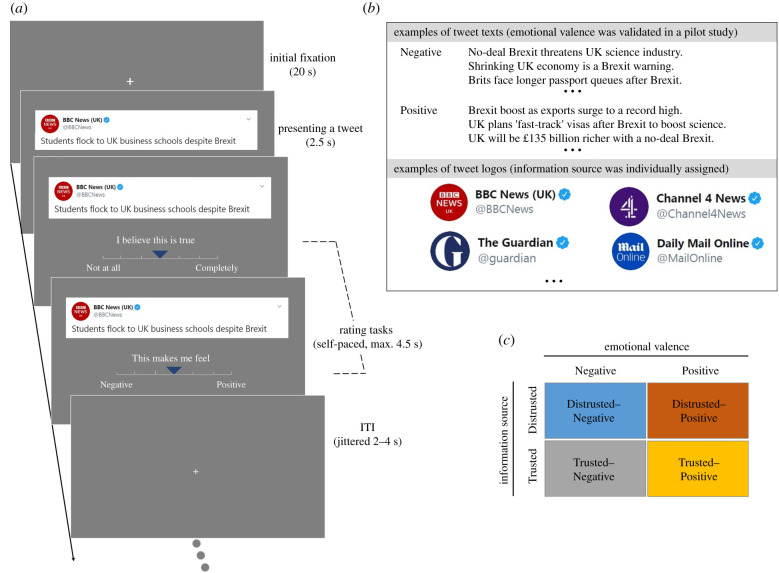
fMRI task and design. (*a*) Schematic of a single trial during fMRI scan. (*b*) Examples of tweet texts and logos. (*c*) A 2 × 2 repeated measures ANOVA design (emotional valence × information source).

Negative valence tweets (i.e. regarding substantial costs/consequences) on Brexit are belief-compatible for Remain voters who oppose Brexit, while Positive valence tweets (i.e. regarding benefits/rewards) are belief-incompatible. These factors, information source (Trusted, Distrusted) and emotional valence/belief compatibility (Negative/Compatible, Positive/Incompatible), were fully crossed in a 2 × 2 ANOVA design (figure 1*b,c*).

### (c) Task and fMRI design

Participants completed three practice trials before confirming that they understood the task. We used a rapid-event-related design. During the task a fixation cross was shown for the initial 20 s. The tweets and accompanying logos were presented for 2.5 s; two rating tasks followed, measuring belief and feeling, counterbalanced across participants. For the belief rating task, the statement ‘I believe this is true’ was presented with a 7-point scale from ‘Not at all (1)’ to ‘Completely (7)’ (reversed for 50% of participants). For feeling ratings, the phrase ‘This makes me feel’ was presented with a 7-point scale from ‘Negative (1)’ to ‘Positive (7)’ (reversed for 50% of participants). Cursor position on the scales always initiated in the middle. Inter-trial interval (ITI) was jittered 2–4 s. Participants used left and right thumb buttons to move the cursor to the left and to the right, respectively, on the response scales, and pressed the right index button to finalize their response (figure 1).

### (d) fMRI apparatus and MRI data acquisition

Participants viewed the visual stimuli via a mirror attached on a head coil; we used PsychoPy (v. 3) to present stimuli [24]. Two response grips were used (NordicNeuroLab). We acquired imaging data of high resolution T1-weighted three-dimensional anatomical images (TR 2500 ms, TE 4.37 ms, 256 × 256 mm 100% field of view (FOV), 1 mm slice thickness), gradient-echo fieldmaps (same slice as the echo-planar imaging (EPI) images, resolution 3 × 3 mm, TR 599 ms, TE_1_ 5.19 ms, TE_2_ 7.65 ms, flip angle 60°, bandwidth 260, TA 1 m in 15 s), and gradient EPI functional images (TR 2260 ms, TE 27 ms, 192 mm 100% FOV, in-plane resolution 3 × 3 mm, 3 mm slice thickness, no gap, flip angle 80°, slice order = Siemens ascending interleaved), using a 3 T Siemens Skyra MRI scanner (Siemens Healthineers, Erlangen, Germany).

### (e) fMRI data analysis

fMRI data were analysed using Statistical Parametric Mapping (SPM12 (v 7771), Wellcome Trust Centre for Neuroimaging, London, UK) and MATLAB 2019b (MathWorks). In pre-processing, the first two functional images were discarded to ensure T1 saturation. Remaining functional images were corrected for slice-timing, realigned, distortion-corrected via gradient fieldmap (Realign & Unwarp of SPM12 using a voxel displacement map, [25,26]) and co-registered to the corresponding individual T1-weighted anatomical images. The anatomical images were segmented, with parameters used to normalize functional images with the Montreal Neurological Institute (MNI) brain template. Normalized functional data were smoothed with a three-dimensional isotropic Gaussian kernel 6 mm full width at half maximum. For statistical processing, the general linear model (GLM) was used at first-level analysis. Presentation of tweet, rating of belief score and rating of feeling score were modelled as events for each condition (2 levels of 2 factors) using the canonical haemodynamic response function (HRF). Additional regressors of no interest included the temporal derivative of each GLM predictor, high-pass filter (128 s) regressors and six rigid body transformation parameters from spatial realignment. Contrasts of parameter estimates (greater than fixation (not explicitly modelled)) of each event were used to compute contrast images at the first level, which were then entered into the second-level GLM model of a two-way repeated measures (2 × 2) flexible factorial ANOVA for the whole brain. We performed separate ANOVAs during the first presentation of a tweet, belief rating task, and feeling rating task. Statistical threshold was set at *p* < 0.001 (uncorrected) at the voxel level, and with a cluster-level extent threshold of *p* < 0.05 (false discovery rate, FDR) applied for multiple corrections. Cluster parameter estimates included all voxels, extracted using the Marsbar toolbox [27].

## 3. Results

### (a) Behavioural results

Rating scores and reaction times (RTs) collected during fMRI scans were standardized and analysed using R (v. 4.0.2), brms (v. 2.13.5) and STAN. All regression models sampled from four chains with 1000 trial burn-in and 6000 iterations each, using regularizing, weakly informative Gaussian priors (*µ* = 0, *σ* = 1). All chains converged as indicated by both caterpillar plots and $\hat{R}$ estimates (all = 1.00). Intervals are 95% highest posterior density intervals (HDI) unless otherwise stated.

As belief and feeling ratings were negatively correlated, *r* = −0.19, HDI = [−0.22, −0.16], we performed a 2 × 2 repeated measures Bayesian multilevel multivariate regression, with fixed effects of information source (Trusted, Distrusted) and valence (Negative, Positive) and random intercepts for participants and stimuli. Trusted sources increased belief ratings, *b* = 0.22, HDI = [0.15, 0.29], but did not appreciably affect feeling ratings, *b* = 0.01, HDI = [−0.05, 0.07]. Positive valence/Belief-Incompatible information decreased belief ratings, *b* = −0.79, HDI = [−0.97, −0.61], and increased positive feeling ratings, *b* = 1.17, HDI = [1.05, 1.29]. There was no meaningful interaction for belief ratings, *b* = −0.01, HDI = [−0.10, 0.09], nor for feeling ratings, *b* = 0.04, HDI = [−0.05, 0.12].

Since RTs were also correlated (*r* *=* *0*.04, HDI = [0.01, 0.07]), we repeated the above analysis. Predicting belief RTs revealed negligible effects of Trusted sources, *b* = −0.04, HDI = [−0.12, 0.04], Positive valence/Belief-Incompatible information, *b* = −0.04, HDI = [−0.15, 0.05], and no interaction, *b* = 0.05, HDI = [−0.07, 0.16]. For feeling RTs, the pattern was similar to source, *b* = −0.06, HDI = [−0.14, 0.02], Positive valence/Belief Incompatibility, *b* = 0.10, HDI = [−0.01, 0.19], and the interaction, *b* = 0.11, HDI = [−0.01, 0.20], though the latter two effects provide some suggestion that participants took slightly longer to give feeling ratings to Belief-Incompatible information, particularly when it came from a Trusted source (for descriptive statistics, see supplementary material, table S2).

### (b) fMRI results

**Figure 2.**
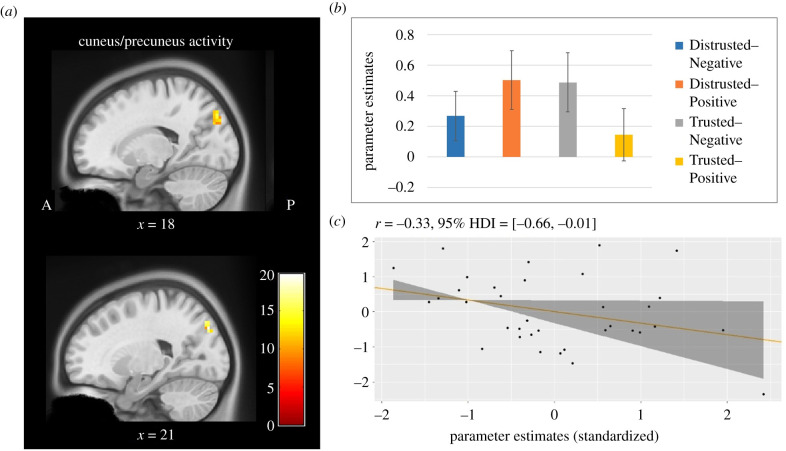
Significant interaction between emotional valence and information source during the belief rating task (*a*) Cuneus and precuneus activity in the right hemisphere. (*b*) Parameter estimates of the cuneus/precuneus activity (error bars denote ±s.e.m.). (*c*) Correlation (and 95% HDI) between parameter estimates and belief ratings (Distrusted–Positive).

A two-way repeated measures ANOVA of whole brain during the belief rating task showed a significant main effect of the information source in the middle occipital gyrus (MOG) of the right hemisphere (table 1, MNI coordinates: *x* = 48, *y* = −76, *z* = −4; *Z*-score = 4.41), and a significant interaction in the cuneus and precuneus in the right hemisphere (figure 2*a*, MNI coordinates: *x* = 21, *y* = −82, *z* = 38; *Z*-score = 4.11), where the parameter estimates of Distrusted–Positive and Trusted–Negative were significantly greater than Distrusted–Negative and Trusted–Positive, respectively (figure 2*b*). There was a negative correlation, *r* = −0.33, HDI = [−0.66, −0.01], between parameter estimates in the cuneus/precuneus and belief ratings for Belief-Incompatible information from a Distrusted source (i.e. Distrusted source–Positive valence; figure 2*c*), but no equivalent result for compatible information from a Trusted source, *r* = 0.12, HDI = [−0.22, 0.48].

**Table 1.** fMRI ANOVA results during the belief rating task. R, right hemisphere.

brain regions	MNI coordinates			*Z*-score	cluster size (k)
	*x*	*y*	*z*		
main effect of information source					
R middle occipital gyrus	48	−76	−4	4.41	21
interaction: information source × emotional valence					
R cuneus and precuneus	21	−82	38	4.11	34

**Figure 3.**
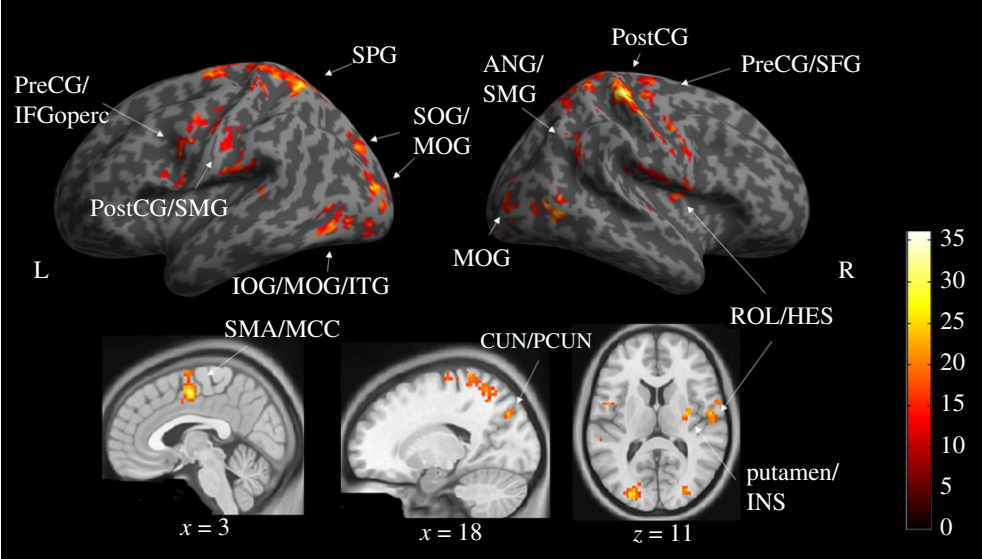
The main effect of emotional valence on neural activation during the feeling rating task (see also electronic supplementary material, table S3 and figure S1). PreCG, precentral gyrus; IFGoperc, inferior frontal gyrus, opercular part; PostCG, postcentral gyrus; SMG, supramarginal gyrus; SMA, supplementary motor area; MCC, middle cingulate gyrus; ROL, rolandic operculum; HES, heschl's gyrus; SPG, superior parietal gyrus; SOG, superior occipital gyrus; IOG, inferior occipital gyrus; ITG, inferior temporal gyrus; MTG, middle temporal gyrus; MOG, middle occipital gyrus; ANG, angular gyrus; INS, insula; CUN, cuneus; PCUN, precuneus.

An identical analysis on the feeling rating task showed significant activations in the main effect of valence/belief compatibility across fronto-parietal–occipital lobes (figure 3, electronic supplementary material, table S3 and figure S1), which are largely clustered and include bilateral superior/middle/inferior occipital gyrus, bilateral postcentral gyrus, superior frontal gyrus, superior parietal gyrus, supplementary motor areas extending to middle cingulate gyrus, right putamen/insula, right Rolandic operulum extending to Heschl's gyrus, right precentral gyrus extending to superior fontal gyrus, angular gyrus and supramarginal gyrus. There was no significant activation for the main effect of information source nor interaction.

## 4. Discussion

Our participants rated their belief in, and feelings about, politically salient tweets. Behaviourally both (dis)trust in information source and emotional valence/belief compatibility affected belief ratings, but only valence/belief compatibility affected feeling ratings. RTs were slightly longer when rating feelings about Positive valence/Belief-Incompatible tweets. The fMRI analyses of the feeling rating task revealed only a main effect of valence/belief compatibility in a wide network with clustered regions related to multiple cognitive processes, including language processing in the frontal, temporal and parietal regions, motor movements in the precentral and postcentral gyrus, and visual processing in the occipital areas in both hemispheres. By contrast, when rating *belief* in our political tweets, there was a significant main effect of information source in right MOG and a significant interaction effect in right cuneus and precuneus. That interaction effect, driven by the negative correlation with belief ratings for Distrusted Positive-valenced stimuli, suggests additional processing occurred for information matching source expectations (i.e. Distrusted sources providing Belief-Incompatible information).

While these results are limited, and somewhat unexpected given our initial hypotheses centred on PFC activity, they seem most compatible with the I-bH [16] and SR accounts [10,28,29] of misinformation acceptance. Emotion and attention are known to heighten sensitivity to visual cues [30], and recent neurocomputational work suggests that emotion-related activity in visual cortex, such as we found in the belief rating task, may represent an interface of sensory representations of the environment and previous knowledge [31]. Encoding of high-arousal negative information is associated with activity in the MOG, an area that may cooperate with closely related areas (e.g. posterior fusiform and inferior occipital gyrus) in a network specializing in identifying emotionally important visual clues [32]; for example, both MOG and cuneus activity are involved in inferring threat from non-facial cues/body language [33]. Given the identity-relevant nature of the information presented in our study, the activation we observe in MOG and cuneus is also consistent with middle occipital and cuneus involvement in both judgements and attitudes about others and the self [34,35]. Similarly, in close parallel to our findings, the cuneus is involved in information processing when invalid cues appear (i.e. when information is detected to be misleading/wrong) and even more so when they are the result of human intention (versus preprogrammed stimuli; [36]), as well as when people disbelieve unpalatable political information relative to non-political information [23]. People often deliberately scrutinize or attack incompatible and/or distrusted information more than they carefully consider appealing belief-compatible information [9], which is the pattern of activation we find in (pre)cuneus (figure 2*b*). The lack of significant PFC activation for belief-compatible information (from any source) seems consistent with this, and inconsistent with a role for careful reasoning in our task, though we cannot draw strong inferences from the lack of an effect in this context. It may well be that our participants, selected for strong Brexit views, were immediately ‘on guard’ when our stimuli were Brexit-related. Such identity-based motivated scrutiny is likely somewhat automatic, with respect to core identity beliefs.

Indeed, this type of motivated rejection effect is also observed when participants refuse to update beliefs about the self or future finances when information is negative/undesirable [37]. Many also reject overwhelming scientific consensus on a variety of topics by positing complex, though absurd, conspiracy theories—essentially attacking the source of unpalatable information (e.g. [38,39]), but typically only when that information presents a challenge to their pre-existing worldview [10]. Such reactions, though relying on some amount of deliberate cognition, are typically not products of systematic reasoning, but rather affective reactivity [40] that is largely effortless [41], though it can sometimes be the result of rational calculation to discredit information or sources that oppose or hinder one's goals [42,43]. Our results suggest that participants integrated information regarding source trustworthiness into their belief ratings, and when confronted with belief-incompatible information from a distrusted source, believed it less, possibly as a function of differentially critical processing.

## 5. Conclusion

Under motivated cognition accounts of misinformation acceptance (SR and I-bH), people can actively incorporate belief-compatible information and critically scrutinize incompatible information [9]. In the latter case, people do not necessarily systematically reason about strengths and weakness of offending information, but rather critique to reject it and protect the self (cf. [44]). Remain and Leave identities, prevalent in the aftermath of the UK's EU referendum, are personally important and cut across traditional party lines, generating affective polarization as intense as that of partisanship across a range of measures [45]. Our results seem to corroborate the view of motivated rejection of belief-incompatible information. Belief incompatibility itself and beliefs about trustworthiness of sources may be combined to motivate rejection of politically offensive information when that information is a threat to a deeply held/self-defining ideological belief.

## Data accessibility

The data are automatically open access as ESRC RCUK project ES/R001901/1 (doi:10.5255/UKDA-SN-854099).
